# Towards applying NMR relaxometry as a diagnostic tool for bone and soft tissue sarcomas: a pilot study

**DOI:** 10.1038/s41598-020-71067-x

**Published:** 2020-08-26

**Authors:** Elzbieta Masiewicz, George P. Ashcroft, David Boddie, Sinclair R. Dundas, Danuta Kruk, Lionel M. Broche

**Affiliations:** 1 Department of Physics and Biophysics, Faculty of Mathematics and Computer Science, University of Warmia and Mazury in Olsztyn, Słoneczna 54, 10-710 Olsztyn, Poland; 2 Department of Orthopaedics, School of Medicine, Medical Sciences and Nutrition, University of Aberdeen, Foresterhill, Aberdeen, AB25 2ZD Scotland, UK; 3 Department of Pathology, School of Medicine, Medical Sciences and Nutrition, University of Aberdeen, Foresterhill, Aberdeen, AB25 2ZD Scotland, UK; 4 Bio-Medical Physics, School of Medicine, Medical Sciences and Nutrition, University of Aberdeen, Foresterhill, Aberdeen, AB25 2ZD Scotland, UK; 5 Present Address: Faculty of Food Sciences, University of Warmia and Mazury in Olsztyn, Michała Oczapowskiego 4, 10-719 Olsztyn, Poland

**Keywords:** Biophysical methods, Molecular biophysics, Bone cancer, Sarcoma, Biomarkers, Structure of solids and liquids, Biological physics, Characterization and analytical techniques

## Abstract

This work explores what Fast Field-Cycling Nuclear Magnetic Resonance (FFC-NMR) relaxometry brings for the study of sarcoma to guide future in vivo analyses of patients. We present the results of an ex vivo pilot study involving 10 cases of biopsy-proven sarcoma and we propose a quantitative method to analyse ^1^H NMR relaxation dispersion profiles based on a model-free approach describing the main dynamical processes in the tissues and assessing the amplitude of the Quadrupole Relaxation Enhancement effects due to ^14^N. This approach showed five distinct groups of dispersion profiles indicating five discrete categories of sarcoma, with differences attributable to microstructure and rigidity. Data from tissues surrounding sarcomas indicated very significant variations with the proximity to tumour, which may be attributed to varying water content but also to tissue remodelling processes due to the sarcoma. This pilot study illustrates the potential of FFC relaxometry for the detection and characterisation of sarcoma.

## Introduction

Magnetic Resonance Imaging (MRI) is a powerful method used in the medical diagnosis of a range of different soft tissue pathologies. The principle of MRI lies in detecting differences in the behaviour of nuclear spins (in most cases from ^1^H) between pathological tissues and their healthy counterparts, which can be exploited as a source of contrast to form images. Spin–lattice and spin–spin relaxation times, denoted as *T*_1_ and *T*_2_ respectively, are two important sources of MRI contrast that describe how fast tissues return to magnetic equilibrium after excitation. To simplify calculations one often uses their reciprocal values, *R*_1_ = *1/T*_1_ and *R*_2_ = *1/T*_2_, which are referred to as spin–lattice and spin–spin relaxation rates, respectively^1–3^ but convey the same information. *R*_1_ usually shows much better contrast at fields below 0.5 T but most clinical scanners operate at 1.5 T or 3 T to achieve high spatial resolution. Paramagnetic contrast agents are commonly used to improve contrast^4,5^, providing relaxation enhancement caused by strong magnetic dipole–dipole interactions between protons (hydrogen nuclei, ^1^H) from the tissues and the paramagnetic centre (typically gadolinium or manganese ions).

Despite the huge progress in advanced contrast agents and MRI technology, the early diagnosis and treatment of patients with musculoskeletal (MSK) malignancies (sarcomas) remains a major challenge. Initial detection of MSK malignancies depends upon clinical examination, fine needle aspiration cytology or core biopsy and MRI. MRI is also used in the follow-up and surveillance of patients with suspected local recurrence following treatment. Unfortunately, the imaging characteristics of tissues using conventional MRI are not diagnostic for a large number of soft tissue tumours and therefore careful multidisciplinary interpretation of the combined results are required in reaching a final diagnosis. Despite this, it can still be challenging to estimate tumour aggressiveness or resection margins.

In this work we investigate sarcoma using Fast Field-Cycling (FFC), an NMR technique measuring *R*_1_ over a broad range of magnetic fields^6,7^. FFC provides unique information on molecular dynamics that can be understood from basic NMR principles^8–10^. FFC methods repeat the measurement of *R*_1_ over a range of magnetic field strengths to obtain a profile of *R*_1_ variations as a function of the proton NMR resonant frequency (${\omega }_{H}$), referred to as a relaxation dispersion profile. This profile is a quantitative measurement of molecular dynamics occurring on timescales of ms to ns within the material under study, providing unique insights on tissue structure non-invasively. NMR relaxometry has been intensively expanding in recent years, offering interesting applications in physics and chemistry for liquid^11–13^, macromolecular (polymers, proteins)^6,11,13–15^ and solid state systems^16–19^, including the possibility of modelling the relaxation enhancement for paramagnetic contrast agents^20–22^. Relaxation dispersion profiles for protein systems also show frequency-specific relaxation maxima, referred to as quadrupole peaks^23–26^, an effect referred to as Quadrupole Relaxation Enhancement (QRE)^16–19,23–28^, the physical origin of which is well understood^16,27,28^. In biological systems QRE is due to the presence of ^14^N nuclei in proteins where motion is restrained as a result of cross-linking or aggregation, for example.

NMR relaxation dispersion studies convey information of particular interest in medicine as it can be expected that pathological changes lead to large modifications of the extra- and intra-cellular environments. This in turn should affect the overall dynamics of water and proteins, which should be quantifiable by *R*_1_ relaxometry. Indeed, pioneering studies^29–31^ have demonstrated that this was the case in certain diseases such as breast cancer and multiple sclerosis but their aim was primarily to optimize tissue contrast at a given fixed field for MRI. More recently, other research groups have investigated how NMR relaxometry can inform on the interactions between water and proteins^24,32,33^. QRE effects have also been observed for tissues and its amplitude has been correlated with the fraction of immobilized proteins^34–36^.

Most importantly for our purpose, great efforts are made to translate FFC to imaging^37,38^ and the research team at the University of Aberdeen has developed two whole-body FFC imaging scanners^39,40^, allowing in-vivo scans of pathologies and unlocking potential applications in biological research and medicine. With more than 100 human scans completed to date (including patients), in vivo FFC imaging of sarcoma is now possible. Motivated by the needs to improve diagnosis technologies for musculoskeletal malignancies and by the new possibilities offered by whole-body FFC imaging, we have investigated *R*_1_ relaxation properties of sarcoma using formaldehyde-fixed resections from surgery and have examined differences in the *R*_1_ relaxation dispersion profiles of musculoskeletal malignancies, tissues adjacent to the tumour and apparently healthy tissues taken at the resection margin, compared with histopathological findings. The purpose of this pilot work was to investigate differences in the dispersion profiles of these tissues and to propose interpretations using the histopathological findings, in preparation for clinical trials identifying relevant ranges of magnetic fields for the FFC imaging of sarcoma.

## Results and analysis

**Figure 1:**
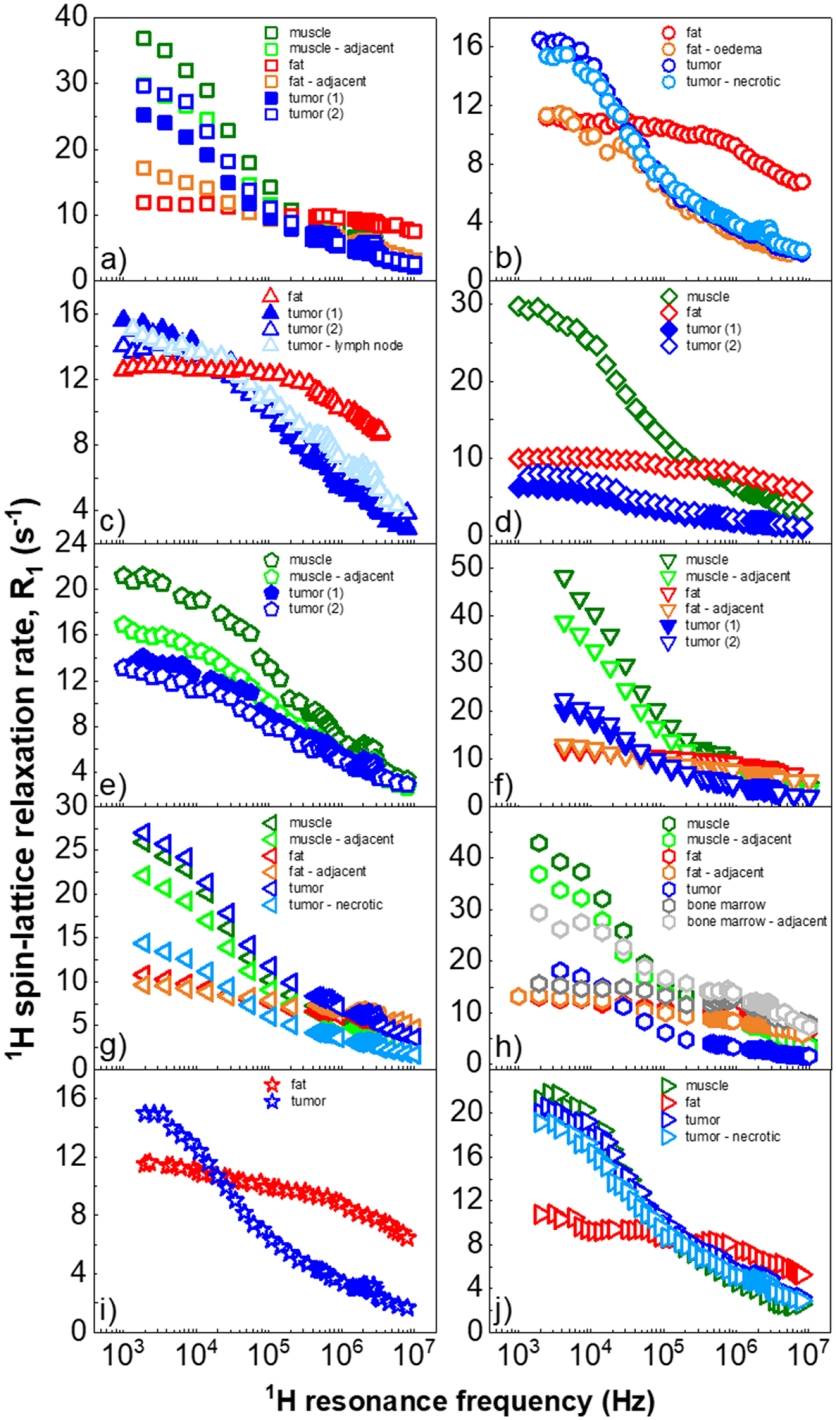
^1^H spin–lattice relaxation dispersion profiles for all the tissue samples, before the scaling procedure. Fatty tissues (in red) show a marked two-segment shape in log–log plots, which clearly differs from all other samples except for oedematous fatty tissues in patients a and b, which appeared closer to tumour profiles. Dispersion profiles from muscle (in green) and tumours (in blue), on the other hand, exhibit similar shapes with quadrupolar peaks around 2.5 MHz and large dispersions below 100 kHz (2.3 mT). Measurement errors were typically between 1 and 4% of *R*_1_.

**Figure 2:**
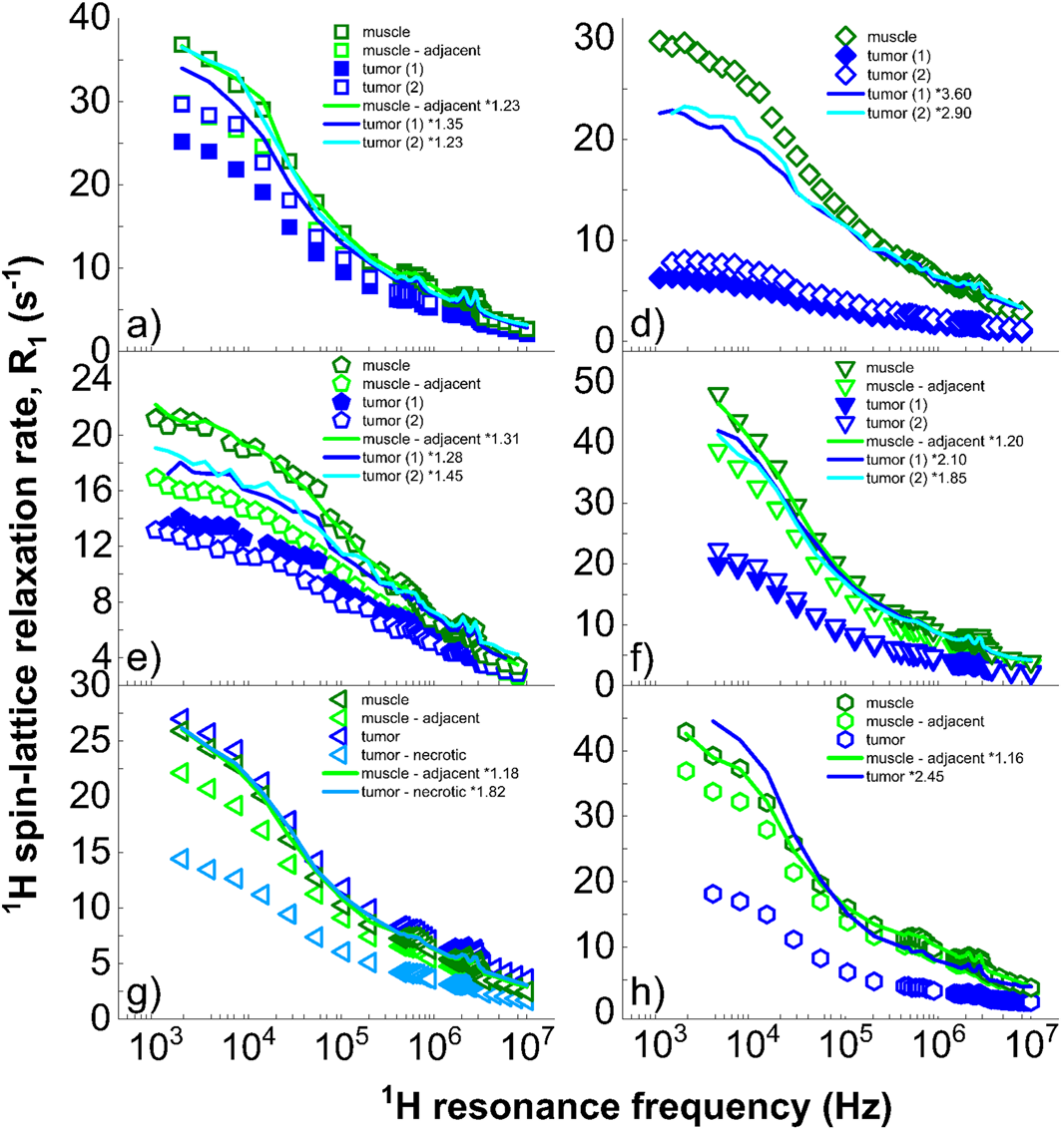
Comparison of *R*_1_ relaxation dispersion profiles for the resections presenting both sarcoma and muscle tissue samples. Each graph presents the raw (markers) and the scaled data (solid lines) to provide an overview of the scaling factors found in each sample (value shown in the legends). In patients a, d, e and h, significant differences appeared at low frequencies after applying the scaling procedure described in the methods. These were particularly large in patients d and e.

**Figure 3:**
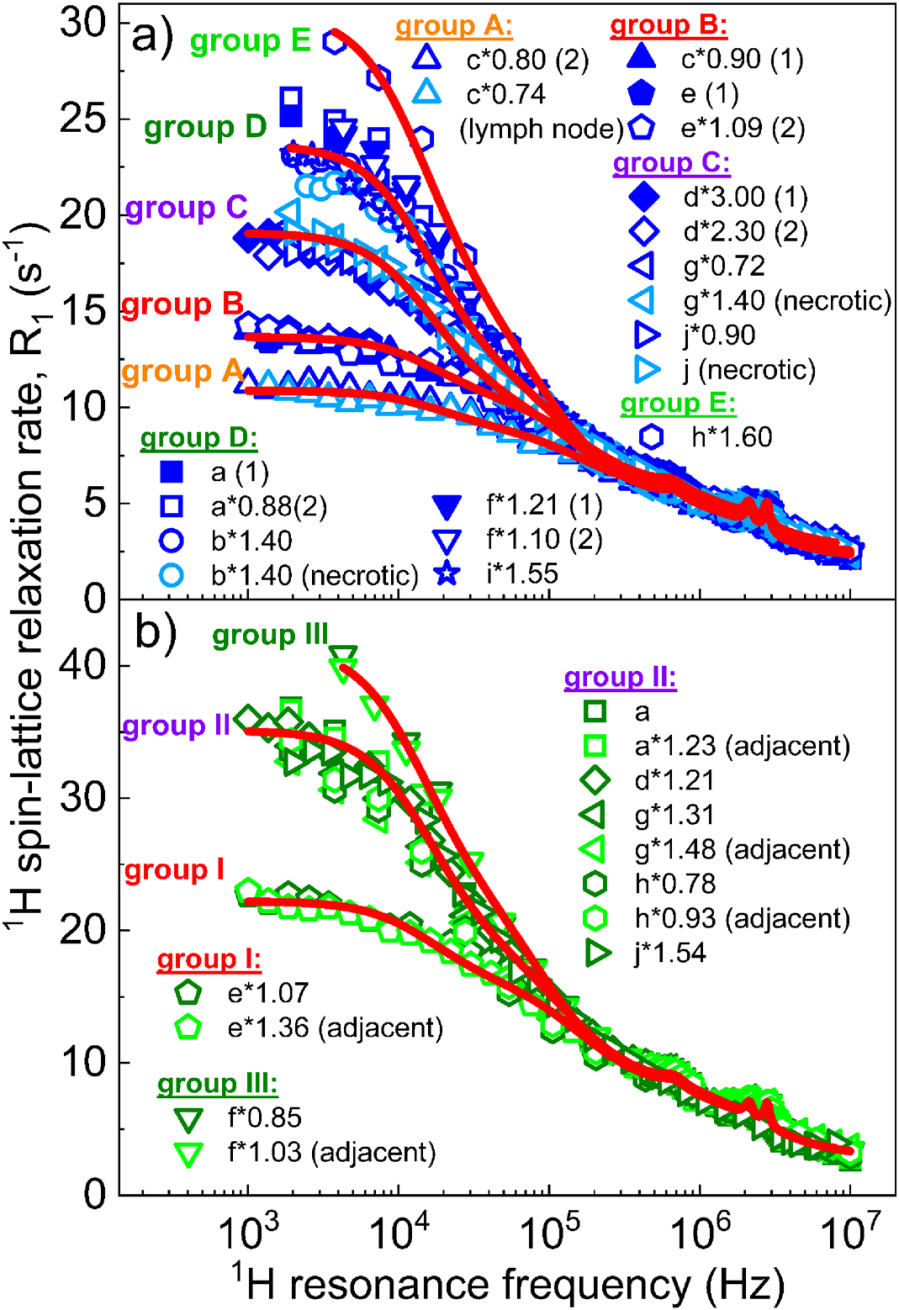
^1^H spin–lattice relaxation dispersion profiles for **(a)** sarcoma and **(b)** muscle tissues, grouped according to the description in the text. Red lines stand for the theoretical fits.

The whole set of ^1^H spin–lattice relaxation data is shown in Fig. 1 and labelled according to Table 2. Note that some resections provided no peritumoural tissues, while some provided additional tissues such as lymph node or bone marrow. The scaled datasets for muscle and tumours are presented in Fig. 2, while Fig. 3 compares all the scaled dispersion profiles from tumours and muscle tissues, separately. The raw dispersion profiles in Fig. 1 show considerable differences below 1 MHz, which appear most clearly in cases where both tumour and muscle tissues were available (patients a, d, e, f, g, h and j, Fig. 2). For these cases it is possible to compare the relaxation dispersion profiles after scaling; the scaling factors used are shown in Fig. 3 and are analysed separately.

Patient a: datasets from adjacent muscle and tumour sample 2 overlapped before scaling and overlapped with the remote muscle profile after scaling. Dataset from tumour sample 1 showed persevering discrepancies in the low frequency range, indicating different molecular dynamics in this region.

Patient b: this patient did not provide muscle samples. Datasets from tumour samples overlapped before scaling, but showed large differences win the dispersion of fatty tissues. Oedematous fat taken near the tumour showed differences below 40 kHz proton NMR frequency, but was otherwise similar at higher fields.

Patient c: this resection provided a benign lymph node, the dispersion of which overlapped with the tumour samples. The dispersion from fatty tissues followed a very different profile.

Patient d: datasets from the tumour coincided after minor scaling but differed considerably from the muscle and fat tissues.

Patient e: datasets from muscle and adjacent muscle overlapped after scaling. So did datasets from the tumour but they did not coincide with the scaled muscle data.

Patient f: Note that data at very low frequencies was not measured due to human error during the protocol, nevertheless a trend is visible. Datasets from muscle and adjacent muscle overlapped after minor scaling. Datasets from the tumour almost overlapped, leaving small discrepancies at low frequencies.

Patient g: all the datasets overlapped after rescaling. Note that the necrotic tumour profile needed a rather large scaling factor of 1.82.

Patient h: the remote and adjacent muscle data coincided after a small rescaling and, once more, exhibited differences at low frequencies with the profile of tumour tissues. In this case discrepancies also showed at high frequencies.

Patient i: this resection did not provide muscle samples, but showed very different profiles from tumour and fat datasets.

Patient j: after scaling, the relaxation profiles for muscle, tumour and necrotic tumour tissues did not show significant discrepancies.

In five out of seven cases we observed clearly different shapes of ^1^H spin–lattice relaxation dispersion profiles for tumour and muscle tissues. The relaxation processes were consistently less efficient in tumour samples, which agrees with findings from clinical MRI. The relaxation dispersion profiles from fatty tissues grossly differed from the others even in the cases of liposarcoma (a) and (b) where histological analyses found solid spindle cells. Resection (b) showed a tumour dispersion profile approaching that of surrounding oedematous fat but differing in the low frequency range, indicating large structural changes in these peritumoural fatty tissues. Similar findings appeared in the relaxation profile of bone marrow adjacent to tumour in (h), with marked differences from healthy fatty tissues in the low-field range while non-involved bone marrow showed a dispersion profile similar to that of healthy fatty tissues. Finally, the relaxation dispersion profile for the benign lymph node (c) resembled the profile for sarcoma for this case.

Following rescaling, the sarcoma dispersion profiles visually fell into five groups that we labelled A to E from the lowest to the highest dispersion rate at the low-end of the magnetic field (Fig. 3a). Group A included one leiomyosarcoma and the benign lymph node from the same patient though another sample from that patient belonged to group B, which also contained the Ewing sarcoma, indicating leiomyosarcoma heterogeneity. Groups C and D were the most commonly observed (13 samples over 19) and shared the dedifferentiated sarcomas and the myxofibrosarcoma, regardless of Trojani grades. Group E included the only case of chondrosarcoma, the dispersion of which was so steep that it stood clearly away from all others. One hypothesis to explain these groupings is the rigidity of the samples: soft liposarcomas and Ewing sarcoma exhibited the lowest low-field dispersion while hard chondrosarcoma showed the highest.

Similarly, the scaling procedure revealed three groups of relaxation profiles in the muscle tissues (Fig. 3b). We numbered them I, II and III with increasing dispersion steepness: group I included a single relaxation dispersion profile from resection (e) (Ewing sarcoma from the cranio-occipital region); group II included most of the muscle tissues investigated in this study; group III only comprised resection f (undifferentiated sarcoma) taken from the deltoid muscle and may have been affected by the pressure due to the large tumour developing.

For tumour dispersion profiles, the presence of myxoid components was found to significantly lower the scaling factor (− 0.71 ± 0.46, p-value < 0.005 from t-test). The scaling factors were also larger for muscle samples adjacent to tumours compared to samples taken away from the tumour by a factor 1.21 ± 0.06 (p-value < 0.001 from t-test), indicating longer relaxation times in peritumoural areas likely due to tissue remodelling. These observations are independent from variations in the overall shape of the dispersion curves, and therefore indicate variations in the weighting of the relaxation processes responsible for the observations made here, possibly due to effects such as dilution with extracellular water.

The model-free parameters obtained from the analysis of the relaxation data for the five groups of pathological tissues are included in Table 1 (details are shown in Supplementary materials, Fig. S1 and S2). As the values of the ^1^H spin–lattice relaxation rates have been scaled, we report the ratios ${C}_{s}^{HH}/{C}_{i}^{HH}$ and ${C}_{s}^{HH}/{C}_{f}^{HH}$ instead of the arbitrary values of the dipolar relaxation constants. The values of the correlation times for slow water motions showed a clear increase from group A to group E, agreeing with the hypothesis that groups are related to rigidity, and the amplitude of the quadrupolar peaks did not change significantly within the three muscle groups but were markedly larger in muscles than in the sarcoma groups (+ 0.61, p-value < 0.01 from t-test), indicating significant differences in protein content and/or structural environment. Differences in peak amplitude may also be seen within sarcoma groups, with groups A and B possibly showing larger peak amplitudes, but the low number of observations makes this observation difficult to support statistically.

**Table 1:** Parameters obtained from the analysis of the groups of ^1^H relaxation dispersion profiles for muscle and pathological tissues.

Fit parameter	Muscles			Sarcomas				
	Group I	Group II	Group III	Group A	Group B	Group C	Group D	Group E
${C}_{s}^{HH}/{C}_{i}^{HH}$ (× 10^–3^)	82.1 (17)	214 (29)	216 (22)	76.6 (25)	81.7 (19)	179 (23)	240 (32)	281 (35)
${C}_{s}^{HH}/{C}_{f}^{HH}$ (× 10^–3^)	9.72 (2.4)	23.3 (3.5)	23.4 (3.2)	6.5 (2.1)	9.3 (2.3)	22.3 (3.8)	29.8 (4.9)	35.4 (5.1)
${\tau }_{s}$ (µs)	5.21 (0.58)	5.36 (0.29)	6.03 (0.35)	4.46 (0.74)	4.90 (0.60)	5.27 (0.22)	5.35 (0.24)	5.88 (0.39)
${\tau }_{i}$ (ns)	530 (64)	640 (74)	879 (70)	516 (87)	524 (69)	527 (54)	610 (73)	899 (102)
${\tau }_{f}$(ns)	38.6 (7.1)	38.9 (5.6)	39.8 (4.1)	39.6 (6.9)	41.0 (7.3)	37.3 (6.4)	41.2 (6.8)	41.4 (5.1)
$A$(s^-1^)	3.12 (0.32)	3.03 (0.24)	3.03 (0.21)	2.72 (0.18)	2.71 (0.19)	2.23 (0.16)	2.18 (0.17)	2.38 (0.09)
${\tau }_{Q}$ (µs)	1.02 (0.26)	1.04 (0.23)	1.07 (0.18)	0.98 (0.22)	1.00 (0.21)	1.00 (0.18)	1.03 (0.15)	1.02 (0.20)
$\eta$	0.42	0.40	0.41	0.42	0.40	0.41	0.41	0.40
${a}_{Q}$ (MHz)	3.25	3.29	3.30	3.26	3.26	3.29	3.29	3.30
Θ (°)	77 (22)	77 (25)	74 (17)	77 (20)	78 (20)	80 (25)	78 (18)	75 (21)
Φ (°)	53 (6)	57 (6)	57 (4)	48 (5)	53 (5)	52 (4)	54 (3)	53 (4)
${r}_{HN }$(Å)	3.27 (0.16)	3.30 (0.14)	3.26 (0.10)	3.57 (0.16)	3.48 (0.13)	3.43 (0.12)	3.26 (0.09)	3.49 (0.14)

## Discussion

Firstly, we wish to stress that we are aware of the large variety of phenotypes in sarcomas and the number of cases analysed here is low. However, the results showed clear groups, sometimes including different cases, which is a very interesting feature for potential characterisation of sarcomas and provides a first insight into the use of FFC relaxometry as a diagnostic tool even if the origin of the differences observed could not be clearly determined to date. It is also important to keep in mind that the tissues were fixed in formaldehyde, which modifies protein dynamics by cross-linking and is known to affect certain tissues^41^. However, observations by our team in muscle and fatty tissues suggests that the effect of formalin on the dispersion profiles of tissues is mild and reproducible, in particular in muscles (see data in Supplementary materials).

The scaling approach led to the identification of five groups of ^1^H spin–lattice relaxation data for sarcomas, characterised by different low-frequency dispersions. The dispersion profiles are closely linked to molecular dynamics, so these correspond to distinct types of dynamics, but their nature is currently unknown. However, it is striking to see that the 19 samples did not exhibit a continuum of possibilities in the low-frequency regime, which could be expected to be a natural outcome from such random processes as tumour biology, but instead did provide clearly-defined categories as if elements that were constitutive of the molecular dynamics of the tissues were present or absent from the environment, or if different types of cell lines co-existed.

Results from the literature^42,43^ showed that transverse relaxation processes for in vivo breast tumour models are mostly due to intracellular relaxation and that the magnetisation propagates to the slowly-relaxing extracellular compartment via transmembrane water exchanges due to metabolic activity. However, in our case the cells were fixed in formaldehyde so the cell metabolism was null and only passive membrane porosity remained for trans-membrane exchanges, which is known to be largely increased by fixation in certain tissues^41^. Therefore, data interpretation is not clear: the categories of sarcoma dispersions observed are either due to distinct types of intra- and extracellular environments, likely due to the expression of different structural proteins, or to their membranes being affected differently by formalin fixation, showing different membrane phenotypes (or both). Moreover, simulations from Xin et al.^44^ warn us to be cautious about the determination of the relative influence of each compartment and more biological information is needed before quantitative biological information can be extracted from the dispersion profiles.

We can however comment that the groups of dispersions observed could be associated with differences in the overall rigidity of the tissues, since rigid systems tend to confine water, leading to slow molecular dynamics and larger dispersion at low frequencies, as observed in the samples. This hypothesis is supported by the chondrosarcoma showing the steepest dispersion, followed by a solid myxofibrosarcoma, while the lowest dispersions appear from a leiomyosarcoma and Ewing sarcoma. The “hardness” of tumours depends on their cellularity, whether they form matrix or not, and the type of matrix. Cancer cells seem to soften with higher grade so that tumour tissue stiffness would be due to the extracellular matrix, but this is still a topic of debate^45^ and may not be true in fixed tissues, where protein-rich intracellular environments may provide tighter gels after cross-linking for cells that are particularly active in protein secretion. In any case, chondrosarcomas form hyaline cartilage matrix and so are the hardest to the touch amongst the tumours listed here, even for low cellularity. Leiomyosarcomas are usually cellular and solid. Ewing sarcomas are formed by relatively fragile cells and do not form matrix and would be expected to be the softest (additionally, the corresponding case from our study had been previously treated and contained necrotic response as well). In general terms, myxofibrosarcomas are expected to be softer than leiomyosarcomas because their myxoid matrix is soft and jelly-like due to its high content of proteoglycan and other polymer components^46^, however they also exhibit variable cellularity and are characterized by alternating low-cellularity myxoid zones and cellular non-myxoid zones. It is not known whether the area sampled from the one myxofibrosarcoma in the series was of myxoid or cellular morphology.

We also observed large variations in the value of the scaling factors between sarcoma samples, which may be explained by differences in water-to-protein content, especially in the extracellular space given the significant link with the presence of myxoid components. Additional bulk water reduces the probability of water-protein interactions, which results in lower effective ${r}_{HH}$ terms in the expression of the ${C}_{f}^{HH}$ factors of Eq. (1) and acts as a homogeneous scaling factor. Note that water content may be modified during the fixation process so our estimations of the scaling factors may have a systematic bias compared with fresh tissues.

The relaxation data from muscle tissues showed three different groups, even though two of these groups only held tissues from a single patient. Such variability in the relaxation features of muscle tissue has not been observed before to the authors’ knowledge and this effect should be further investigated. We hypothesize that this effect may relate to differences in the physiology of the muscles studied, in the orientation of the muscle fibres in the NMR tube or in unusual pathological remodelling processes.

The scaling factors in muscle samples showed a significant increase with proximity to tumour (1.19 + /− 0.07, *p*-value < 0.005 from t-test) possibly indicating a tendency to swell or a modification in the extracellular environment due to remodelling mechanisms at the vicinity of tumours. If this effect shows in vivo, it may be used to better understand cancer processes, to assess tumour margin and potentially to increase the detection threshold. In two cases (g and j) the dispersion profiles from tumours and surrounding muscles did not differ significantly, which may be either attributed to tumours showing similar water dynamics compared to muscles, or muscle being largely affected by the tumour and exhibiting similar dynamics.

The data for fat samples clearly stand out from muscle and tumour samples, even for liposarcomas, in agreement with the typical dispersion profiles for polymer melts since fatty tissues in adults are composed of triglycerides at about 99%^47^. Dispersion profiles of polymers are known to follow power laws with specific values of exponents^6^. However, fatty tissues collected in the vicinity of liposarcomas (patients a and b) showed large differences in their NMR dispersion profiles compared to those collected at the resection margins, indicating strong influences in the local molecular dynamics from the neighbouring tumour. This is of clinical interest since it can be an efficient contrast mechanism to delineate liposarcoma from peritumoural regions and surrounding tissues.

The model-based quantitative analyses bring additional insights. Quadrupolar peaks, as modelled by the ${R}_{1}^{HN}\left({\omega }_{H}\right)$ relaxation contribution in Eq. (2), were not observed in adipose tissues but did appear in muscle tissues and sarcoma, with sarcoma showing significantly lower peak amplitude (*A*) than muscle tissues, indicating alterations of protein matrices in the tumoural environment. The other parameters associated with QRE, namely ${\tau }_{Q}$, ${a}_{Q}$, $\eta$, $\Theta ,\Phi$ and ${r}_{HN}$, did not show significant differences. Comparing with other publications investigating QRE in proteins (bovine serum albumin, albumin from human plasma, elastin and lysozyme)^26^, the quadrupole parameters confirm the current model stating that the QRE are associated with ^14^N nuclei of protein backbones^23,48,49^. In both tissues and proteins the correlation time ${\tau }_{Q}$ characterizing the fluctuations of the ^1^H–^14^N dipole–dipole coupling was about 1 µs within 20% error, independently of the values of the correlation times ${\tau }_{s}$ for tissues, or in solid (dry) proteins investigated in the literature. This may indicate that ${\tau }_{Q}$ mainly reflects the quadrupole relaxation time originating from local fluctuations of the electric field gradient tensor at the ^14^N site, which are not specific. However, the effective ^1^H–^14^N distance for tissues, ${r}_{HN}$, was found to vary between 3.1 and 3.7 Å which is much larger than for solid proteins (1.65–1.7 Å)^26^. This effect may be caused by the presence of water molecules in the vicinity of the protein backbones, possibly increasing the ^1^H to ^14^N ratio involved in the QRE. Moreover, taking into account that the distance between water protons and the ^14^N nuclei of the protein backbones is likely larger than the ^1^H–^14^N distance within the backbone, the effective value of ${r}_{HN}$ could become larger.

The quantitative analysis of the ^1^H spin–lattice relaxation dispersion data suggests that the ^1^H–^1^H relaxation contribution reflects three dynamical processes characterized for the pathological tissues by correlation times in the range of 4.46–5.88 µs, 516–899 ns and 39.6–41.4 ns for the slow, intermediate and fast dynamics, respectively. Sarcoma groups showed slower dynamics with increasing low-field dispersion. The larger dispersion is a combined effect of slower dynamics and a larger contribution of the relaxation process associated with the slow motion, which agrees with the hypothesis that groups are linked to tissue rigidity. The same parameters measured in muscle tissues gave 5.21–6.03 µs, 530–879 ns and 38.6–39.8 ns and, although the number of cases was small, the slowest dynamics (described by the correlation time ${\tau }_{s}$) may be faster in pathological tissues.

To the authors’ knowledge, this is the first example of a thorough, quantitative analysis of relaxation dispersion profiles from cancer tissues. The results certainly show potentially useful features for non-invasive investigation of cancer biology in vivo, but a better understanding is needed of the biological phenomena underlying the features observed. In clinical MRI, *T*_1_ increases (and therefore *R*_1_ decreases) are often associated with oedema, but changes in the shape of the dispersion profiles indicate more profound modifications of the tissue architecture. Additionally, the fact that sarcoma, which appears in a great variety of sub-types, falls into discrete categories in FFC-NMR may be the hallmark of fundamental biological processes and its understanding may provide new insights into cancer development processes.

The potential of FFC-NMR as a new tool for the exploration of cancer also appears in the peritumoural tissues. Fatty tissues in the vicinity of liposarcomas showed deep modification in their dispersion profiles as well as larger scaling factors, a feature shared by some peritumoural muscle samples. Here again, oedema cannot be the only explanation and this may provide quantitative information on tissue remodelling at the interface between tissues and tumours. Such research is currently receiving increasing attention from the research community, and FFC-NMR may bring precious information.

## Conclusions

The observations reported here have been made both in a qualitative way and following a thorough quantitative analysis of the shapes of the relaxation dispersion profiles using a model-free approach based on three dynamical processes in the tissues. The QRE effects observed in tissues are, as expected from the literature^23,48,50–52^, associated with ^14^N nuclei present in protein backbones. This exploratory study has shed light on FFC-NMR as a new source of information in the context of sarcoma, and potentially for oncology and other pathological processes affecting tissue structures in general. Indeed, pioneering works have pointed out to similar conclusions in various pathologies and biological models^24,30,31,33,49^.

The nature of the information provided by FFC-NMR is closely related to molecular dynamics in healthy and pathological tissues and the surprising results found in the scaled dispersions of sarcomas suggest that much is to be learnt from the interpretation of *T*_1_ dispersion profiles of tumours. The samples studied were fixed in formaldehyde and in vivo studied are needed to test our findings in living tissues, but in any case FFC-NMR can bring novel structural information to histopathological analyses and may become an additional tool for the understanding of cancer physiology.

All these findings are also likely to have interesting applications when combined with FFC imaging^40^, especially since they are endogenous and FFC imaging procedures would therefore be non-invasive. Most of the findings were observed at magnetic fields below 2 mT, which is not accessible by current clinical MRI scanners for which spin–lattice (*T*_*1*_) relaxation is dominated by fast and non-specific dynamics so that gadolinium contrast agents are used.

Our results also bring new research questions: what are the biological structures responsible for the categories of sarcoma, and for the modifications observed in the peritumoural region? How do they relate to the correlation times and other parameters from the model-free approach? More generally, do pathological changes in tumours generally lead to different categories defined by pronounced *T*_1_ differences at slow molecular dynamics? If so, could this large source of contrast be exploited for medical applications, potentially using FFC imaging? To answer these questions, one needs to perform magnetic field-dependent relaxation studies over larger cohorts, including different types of sarcoma, and to include detailed analyses of the cellular environment and proteome. We plan to pursue these efforts by observing sarcoma in-vivo in a future clinical trial, to confirm these findings.

## Methods

### Tissue samples

This research was performed in accordance with the Good Clinical Practices and associated UK regulations. Informed consent was obtained from all participants, who were ten patients undergoing surgical removal of a soft tissue sarcoma under the care of NHS Grampian’s Orthopaedic Department. Recruitment was restricted to patients presenting with tumour volume exceeding 10 cm^3^ to obtain sufficient biopsies without compromising the quality of the pathological examination. This study was approved by the North of Scotland Research Ethic Committee under study number 12/NS/0016.

The tissue resections were fixed for at least 24 h in 10% neutral buffered formalin and histological examinations were conducted as per standard practice, then several cores measuring typically 10 × 5 × 5 mm^3^ were taken from the remaining fixed tissues by the supervising musculoskeletal pathologist. These were taken from (i) regions of the tumour, (ii) peritumoural areas directly adjacent to the tumour (when available), and (iii) healthy tissues taken at a distance of at least 3 cm away from the tumour, in a region assessed as non-affected during examination.

Previous experimentation with fresh and formalin-fixed tissues by our group indicated that formalin fixation does not affect the general shape of the dispersion profiles significantly, though we recognise that the results obtain have to be validated on fresh sarcoma samples, which is difficult to obtain without potentially disrupting assessment of surgical margins.

### ^1^H spin-relaxation measurements

^1^H spin–lattice relaxation measurements for the tissue cores were performed at 37 + /− 0.1 °C by FFC-NMR using a commercially available benchtop device (SMARtracer, Stelar S.r.l., Italy). To prevent sample drying during the acquisition, all samples were placed in an inert fluorinated compound (Fluorinert FC 70, Sigma-Aldrich). The pulse sequence consisted of an inversion recovery sequence with CPMG acquisition using 70 evolution fields selected logarithmically between 10 kHz and 10 MHz ^1^H resonance frequency, with greater sampling between 0.4 and 3.5 MHz where QRE effects occur in biological systems, and 8 different evolution times selected logarithmically by the device. The signal measurement parameters resulted in a total experimental time of 30 to 40 min per sample, depending on the *R*_1_ value of the tissue.

### Histology data

The anonymised clinical and histological data of the patients and their tumours biopsies were accessed with the patient’s consent (Table 2). Clinicopathological variables included patient gender, age at diagnosis and anatomical site. Histopathological parameters included morphological classification, UICC staging, grade (Trojani grade for soft tissue tumours^53,54^ based on tumour classification, necrosis and mitotic count; showing the tendency of the tumour to grow aggressively and to spread, the higher the more aggressive), myxoid component (MC) and the presence of inflammation (I).

**Table 2:** Parameters obtained from histopathology examination for the individual tumour samples.

Patient code	Number of tumour samples	Tumour type	Histological grade^a^	pT stage^b^	Anatomical site	Age	Gender	MC	I
a	2	Dedifferentiated liposarcoma	3	3	Right thigh	68	Female	N	N
b	2, with 1 necrotic	Dedifferentiated liposarcoma^c^	3	1	Left proximal leg	78	Female	Y	N
c	2	Leiomyosarcoma	2	2	Right groin	49	Female	N	N
d	2	Undifferentiated pleomorphic sarcoma	3	4	Left leg	54	Male	Y	Y^e^
e	2	Ewing sarcoma^d^	3	3	Cranio-occipital	46	Male	N	N
f	2	Undifferentiated pleomorphic sarcoma	3	3	Deltoid muscle	59	Male	Y	Y
g	2, with 1 necrotic	Undifferentiated pleomorphic sarcoma	3	4	Left proximal thigh	80	Male	N	Y^f^
h	1	Chondrosarcoma	2	2	Sternum	43	Male	N	N
i	1	Myxofibrosarcoma	2	1	Posterior shoulder	61	Female	Y	Y
j	2 (1 necrotic)	Giant cell-rich pleomorphic sarcoma	2	2	Pelvis	79	Female	N	Y^f^

^a^Trojani grade for soft tissue tumours. Ewing sarcoma high grade 3 by definition.

^b^UICC TNM8.

^c^Including heterologous rhabdomyosarcomatous differentiation.

^d^CD99/ EWSR1 translocation positive.

^e^Peri-tumoural muscle was inflamed.

^f^Haemosiderin deposition.

### FFC-NMR data analysis and comparisons with histology

Fatty tissues showed dispersion profiles vastly different to those of other tissues, so detailed comparisons were only made between muscle tissues and tumours. The shape of the *R*_1_ dispersion profiles is determined by all the molecular dynamics within the tissues, with slow motions affecting the low-frequency end of the profile and vice-versa. This includes bulk water within the tissues, which affects the amplitude of the dispersion profile and is variable between patients, making direct comparison difficult. To avoid this problem the dispersion curves were scaled to a reference, arbitrarily chosen as being the first sample of the study. The scaling procedure was performed by adjusting the part of the dispersion profiles above 4 MHz so that it minimised the sum of squares of the difference with the reference profile. This frequency range was chosen because the high-frequency domain is known to be largely dominated by fast dynamics of internal groups or/and with the presence of the paramagnetic centres, both of which are less affected by changes in tissue structures so that this range is therefore more likely to indicate modifications solely due to water content^30,33^. After scaling, the dispersion curves showed clear categories and were grouped manually. Quantitative comparisons were then performed for each group. Statistical analyses were performed using Matlab 2019a (Mathworks, Natick, MA).

### Theoretical model for the analysis of the dispersion profiles

^1^H spin–lattice relaxation in tissues, ${R}_{1}({\omega }_{H})$, originates from ^1^H–^1^H and ^1^H–^14^N dipole–dipole interactions giving two contributions, ${R}_{1}\left({\omega }_{H}\right)={R}_{1}^{HH}\left({\omega }_{H}\right)+{R}_{1}^{HN}\left({\omega }_{H}\right)$, where ${R}_{1}^{HN}\left({\omega }_{H}\right)$ models the QRE and ${R}_{1}^{HH}\left({\omega }_{H}\right)$ the spin–lattice interactions between protons. Because of the molecular complexity of tissues we applied a “model-free” approach to ${R}_{1}^{HH}\left({\omega }_{H}\right)$, which consists of decomposing the relaxation contributions into a sum of Lorentzian functions to model the free isotropic and independent motions^13,26^. Other similar approaches exist that aimed at modelling complex water-protein relaxation^55,56^, but relaxation processes in tissues are even more complex so we used three terms associated with dynamical processes of different time scales (slow, intermediate and fast), as this was the simplest model with minimal numbers of parameters leading to a good agreement with the experimental data in terms of reduction of the residuals. It is therefore a largely simplifying model that provides a first insight into an otherwise very complex system:

1 $${R}_{1}^{HH}\left({\omega }_{H}\right)={C}_{s}^{HH}\left(\frac{{\tau }_{s}}{1+{\omega }_{H}^{2}{\tau }_{s}^{2}}+\frac{4{\tau }_{s}}{1+4{\omega }_{H}^{2}{\tau }_{s}^{2}}\right)+{C}_{i}^{HH}\left(\frac{{\tau }_{i}}{1+{\omega }_{H}^{2}{\tau }_{i}^{2}}+\frac{4{\tau }_{i}}{1+4{\omega }_{H}^{2}{\tau }_{i}^{2}}\right)+{C}_{f}^{HH}\left(\frac{{\tau }_{f}}{1+{\omega }_{H}^{2}{\tau }_{f}^{2}}+\frac{4{\tau }_{f}}{1+4{\omega }_{H}^{2}{\tau }_{f}^{2}}\right)+A$$

where ${\tau }_{s},{\tau }_{i}$ and ${\tau }_{f}$ are the correlation times associated with the slow, intermediate and fast dynamics and ${C}_{s}^{HH},{C}_{i}^{HH}$ and ${C}_{f}^{HH}$ denote the corresponding dipolar relaxation constants defined as ${C}^{HH}=\frac{3}{10}{\left(\frac{{\mu }_{0}}{4\pi }\frac{{\gamma }_{H}^{2}\hslash }{{r}_{HH}^{3}}\right)}^{2}$, where ${r}_{HH}$ is an effective inter-spin distance accounting for dipole–dipole interactions between several pairs of protons, ${\mu }_{0}$ is the magnetic permittivity of vacuum and $\hslash$ is the reduced Plank constant. The constant $A$ describes relaxation contributions with time scale of 10^-9^ s or shorter which in practice appear independent of ${\omega }_{H}$.

The QRE term can be expressed as^57^:

2 $${R}_{1}^{HN}\left({\omega }_{H}\right)=\frac{2}{3}{\left(\frac{{\mu }_{0}}{4\pi }\frac{{\gamma }_{H}{\gamma }_{N}\hslash }{{r}_{HN}^{3}}\right)}^{2}\times \left[\begin{array}{c}\left(\frac{1}{3}+{\mathit{sin}}^{2}\Theta {\mathit{cos}}^{2}\Phi \right)\left(\frac{{\tau }_{Q}}{1+{\left({\omega }_{H}-{\omega }_{-}\right)}^{2}{\tau }_{Q}^{2}}+\frac{{\tau }_{Q}}{1+{\left({\omega }_{H}+{\omega }_{-}\right)}^{2}{\tau }_{Q}^{2}}\right)+\\ \left(\frac{1}{3}+{\mathit{sin}}^{2}\Theta {\mathit{sin}}^{2}\Phi \right)\left(\frac{{\tau }_{Q}}{1+{\left({\omega }_{H}-{\omega }_{+}\right)}^{2}{\tau }_{Q}^{2}}+\frac{{\tau }_{Q}}{1+{\left({\omega }_{H}+{\omega }_{+}\right)}^{2}{\tau }_{Q}^{2}}\right)+\\ \left(\frac{1}{3}+{\mathit{cos}}^{2}\Theta \right)\left(\frac{{\tau }_{Q}}{1+{\left({\omega }_{H}-{\omega }_{0}\right)}^{2}{\tau }_{Q}^{2}}+\frac{{\tau }_{Q}}{1+{\left({\omega }_{H}+{\omega }_{0}\right)}^{2}{\tau }_{Q}^{2}}\right)\end{array}\right]$$

where the angles $\Theta$ and $\Phi$ describe the orientation of the ^1^H–^14^N dipole–dipole axis with respect to the principal axis system of the electric field gradient at the position of ^14^N, ${\tau }_{Q}$ is the correlation time of the coupling, ${r}_{HN}$ denotes the ^1^H–^14^N inter-spin distance, and ${\gamma }_{N}$ is the ^14^N gyromagnetic factor. At such magnetic fields the energy levels of ^14^N are fully determined by the quadrupole coupling and given as: ${E}_{1}=\frac{1}{4}{a}_{Q}(1-\eta )$, ${E}_{2}=-\frac{1}{2}{a}_{Q}$, ${E}_{3}=\frac{1}{4}{a}_{Q}(1+\eta )$ where ${a}_{Q}$ and $\eta$ denote the amplitude and the asymmetry parameter of the quadrupole coupling, respectively. The amplitude is defined as: ${a}_{Q}={e}^{2}qQ/h$, where $Q$ denotes the quadrupolar moment of the nucleus, while $q$ is the $zz$ component of the electric field gradient tensor. The energy level structure leads to the three transition frequencies: ${\nu }_{-}=\frac{{\omega }_{-}}{2\pi }=\frac{3}{4}{a}_{Q}\left(1-\frac{\eta }{3}\right)$, ${\nu }_{+}=\frac{{\omega }_{+}}{2\pi }=\frac{3}{4}{a}_{Q}\left(1+\frac{\eta }{3}\right)$ and ${\nu }_{0}={\nu }_{+}-{\nu }_{-}=\frac{{\omega }_{0}}{2\pi }=\frac{1}{2}\eta {a}_{Q}$. When ${\omega }_{H}$ matches one of these transition frequencies, ${R}_{1}^{HN}\left({\omega }_{H}\right)$ reaches a maximum.

## Supplementary information

Supplementary Information.
