# Supplementary material for: Towards applying NMR relaxometry as a diagnostic tool for bone and soft tissue sarcomas: a pilot study
Source: Sci Rep. 2020 Aug 26;10:14207. doi: 10.1038/s41598-020-71067-x (PMC7449965; doi:10.1038/s41598-020-71067-x)
Supplement: Supplementary file 1 — Supplementary Information. [file 41598_2020_71067_MOESM1_ESM.docx]

**Towards applying NMR relaxometry as a diagnostic tool for bone and soft tissue sarcomas – a pilot study.**

Supplementary Information

Elzbieta Masiewicz, George P. Ashcroft, David Boddie, Sinclair R Dundas, Danuta Kruk, Lionel M. Broche

**Decomposition of ^1^H spin-lattice relaxation dispersion profiles into individual contributions:**

The two figures below present the details of the decomposition of the *R*_1_ dispersion profiles for the groups of sarcomas and muscle samples, as detailed in the manuscript.


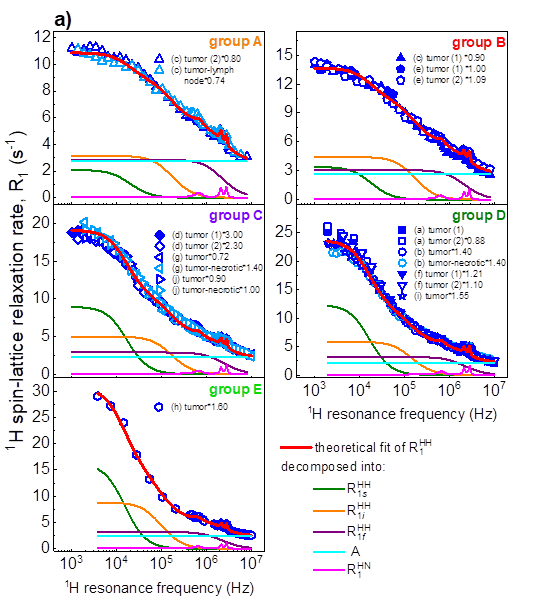


**Supplementary Figure S1.** ^1^H spin-lattice relaxation dispersion profiles, $R_{1}^{HH}$ for sarcoma tissues, grouped according to the description in the text. Red lines – theoretical fits decomposed into the individual relaxation contributions: $R_{1,s}^{HH}$(green lines), $R_{1,i}^{HH}$(orange lines), $R_{1,f}^{HH}$(purple lines), $A$ (light blue lines) and $R_{1}^{HN}$(magenta lines).


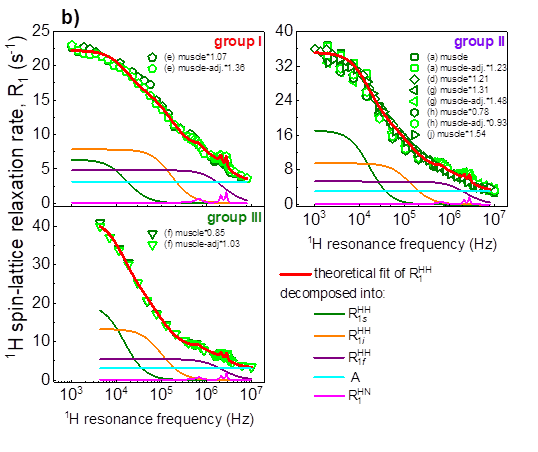


**Supplementary Figure S2.** ^1^H spin-lattice relaxation dispersion profiles, $R_{1}^{HH}$ for muscle samples, grouped according to the description in the text. Red lines – theoretical fits decomposed into the individual relaxation contributions: $R_{1,s}^{HH}$(green lines), $R_{1,i}^{HH}$(orange lines), $R_{1,f}^{HH}$(purple lines), $A$ (light blue lines) and $R_{1}^{HN}$(magenta lines).

**Effect of formaldehyde fixation on fatty and muscular tissues**

The results presented below come from experimentations on animal tissues that could be collected from supermarket supplies or from other studies in an effort to recycle biological material in accordance with the 3 R principles (NC3Rs organization, UK). These were performed to assess the effect of formaldehyde fixation on biological tissues. At the time of writing it has not yet been possible to investigate fresh sarcoma tissues because of the possible adverse effects could have on the clinical procedures followed for the analysis of tissue resections.


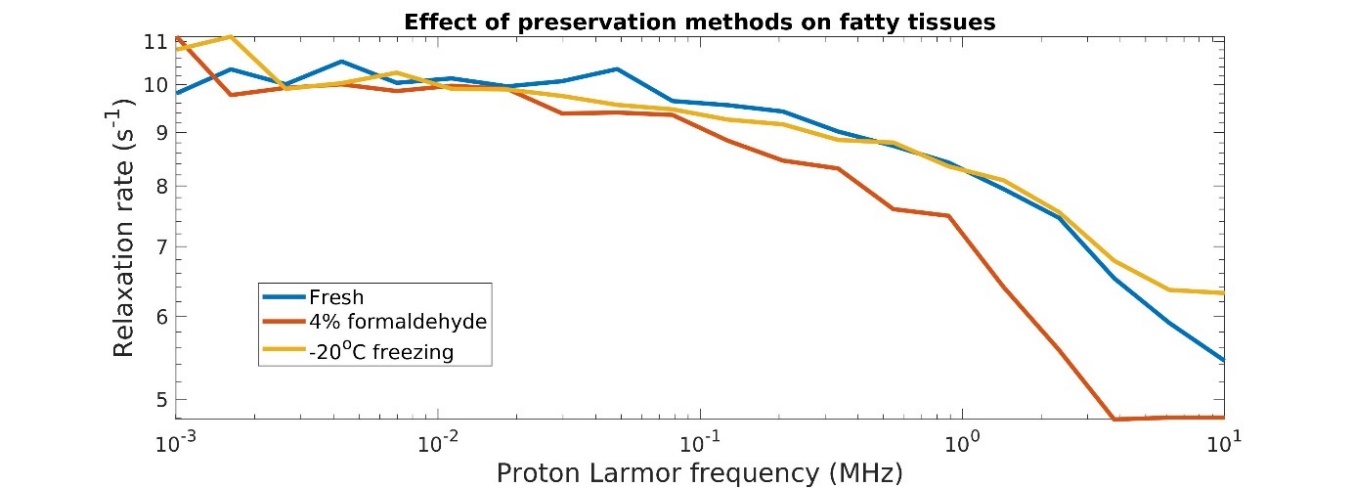


**Supplementary figure S3.** NMRD profiles of white adipose tissues from murine, either fresh (bluie line), exposed to -20^o^C for one day (yellow line) or fixed in 4% formaldehyde for one day (red line). Each curve is an average of 9 measurements. Freezing appears to preserve the dispersion profile but formaldehyde fixation affected the dispersion profile above 100 kHz.


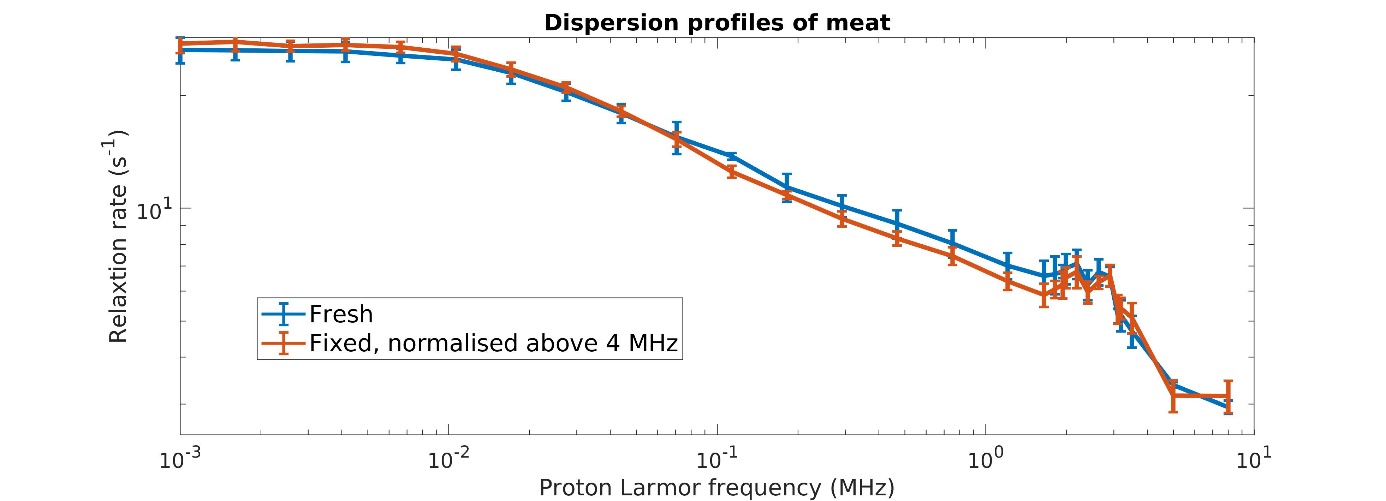


**Supplementary figure S4.** NMRD profiles of lamb meat, either fresh (blue line) or fixed in 4% formaldehyde for one day (red line, normalized to the fresh sample using the procedure described in the Methods section). Each measurement was the average from three samples. One can see that formaldehyde fixation does not have a noticeable effect on the dispersion curves of muscle tissues, with a possible exception of a 20% decrease of R_1_ between 100 kHz and 1 MHz. The amplitude of the quadrupolar peaks was not significantly modified either.

**Data analysis: estimation of *R*_1_ from the magnetization curves**





**Supplementary figure S5:** typical inversion recovery magnetization data obtained from muscle (left) and sarcoma (right) samples from patient a, together with the fitted monoexponential model. This provides an overview of the quality of the data and typical error bars for *R*_1_ values.
